# Supplementary material for: Reconfiguration of quantum states in 𝒫𝒯-symmetric quasi-one-dimensional lattices
Source: Sci Rep. 2017 Aug 18;7:8746. doi: 10.1038/s41598-017-09410-y (PMC5562901; doi:10.1038/s41598-017-09410-y)
Supplement: Supplementary file 1 — Supplementary Information [file 41598_2017_9410_MOESM1_ESM.pdf]

Supplementary Information for reconfiguration of quantum states in $\mathcal{PT}$-symmetric quasi-one-dimensional lattices

Jung-Wan Ryu, Nojoon Myoung, and Hee Chul Park
Center for Theoretical Physics of Complex Systems, Institute for Basic Science, Daejeon 34051, South Korea
(Dated: July 19, 2017)

I. HAMILTONIAN OF A CROSS-STITCH LATTICE

The Hamiltonian of the cross-stitch lattice shown in Fig. 1 is given by

$$E\Psi_j = H_0\Psi_j + H_1\Psi_{j+1} + H_1^{+}\Psi_{j-1}, \tag{S1}$$

where

$$H_0 = \begin{pmatrix} \epsilon_a & -t \\ -t & \epsilon_b \end{pmatrix}, H_1 = \begin{pmatrix} -d & -d \\ -d & -d \end{pmatrix}, \tag{S2}$$

and $\Psi_j = (\phi_j^a, \phi_j^b)^T$. We can set $\Psi_{j+1} = \Psi_j e^{ik}$ and $\Psi_{j-1} = \Psi_j e^{-ik}$ due to the translational symmetry of the unit cells. Finally,

$$H = \begin{pmatrix} \epsilon_a - 2d\cos k & -t - 2d\cos k \\ -t - 2d\cos k & \epsilon_b - 2d\cos k \end{pmatrix}. \tag{S3}$$

Solving the eigenproblem of H when $\epsilon_a = \epsilon_b = 0$ and $t = d = 1$, we obtain the band structure for the cross-stitch lattice in Fig. 2 (a) as follows

$$\varepsilon(k) = -t - 4d\cos k,\ \varepsilon_{FB} = t. \tag{S4}$$

We set $\epsilon_a = \delta/2 + i\gamma/2$ and $\epsilon_b = -\delta/2 - i\gamma/2$. Figure S1 (a) shows a phase diagram of a cross-stitch lattice with real value perturbations to on-site energies in (δ, ε) space when $\gamma = 0$. As δ increases, the size of the energy bandgap increases.

II. TRANSMISSION PROBABILITY OF S-MATRIX IN FINITE SIZED CROSS-STITCH LATTICES

We now discuss the transport problem in finite sized cross-stitch lattices. The system has a cross-stitch lattice with N unit cells as shown in Fig. 1, with two leads connected to both a- and b-sites of the left and right end unit cells of the lattice. The Hamiltonian of this system is given by

$$H = H_{cs} + H_{lead} + H_{coupling}, \tag{S5}$$

where H_{cs}, H_{lead}, and $H_{coupling}$ describe the cross-stitch lattice, leads, and coupling between the lattice and leads, respectively.

$$H_{cs} = \sum_{i=1}^{N} H_0 d_i^\dagger d_i + \sum_{i=1}^{N-1}(H_1 d_{i+1}^\dagger d_i + h.c.) \tag{S6}$$

$$H_{lead} = -\frac{V_0}{2}\sum_{j\neq 0}(c_{j+1}^\dagger c_j + h.c.) \tag{S7}$$

$$H_{coupling} = -g(d_1^\dagger c_{-1} + c_1^\dagger d_N + h.c.), \tag{S8}$$

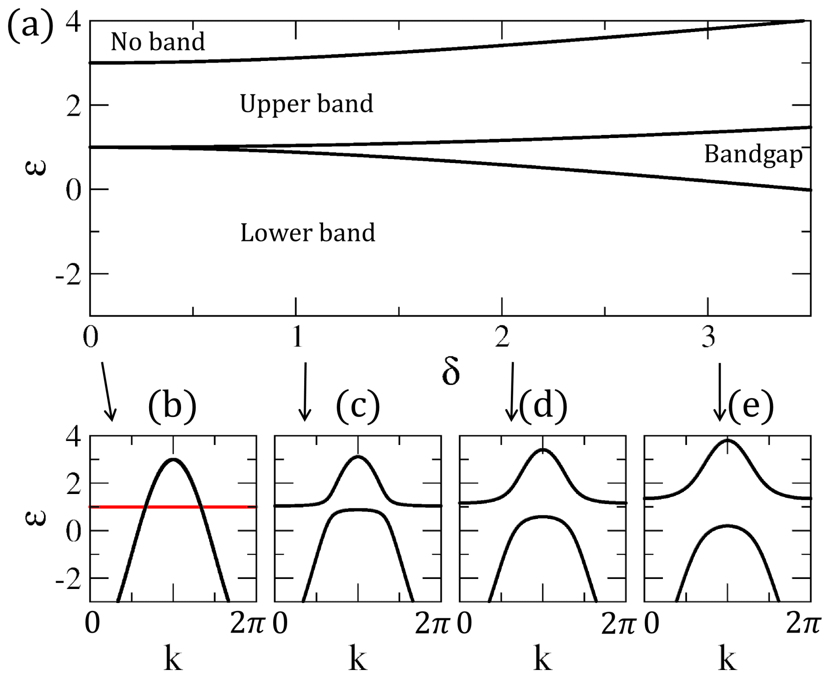

FIG. S1: (a) Phase diagram of a cross-stitch lattice with real value perturbations to on-site energies. The real energy on a-sites (red dots) and b-sites (blue dots) equal $+\delta/2$ and $-\delta/2$, respectively. Black curves represent the boundaries of the energy bands. There are no electronic states in no-band regions as well as bandgap regions. (b)-(d) Energy bands when δ equals 0, 1, 2, and 3, respectively. The red constant energy band in (b) is the flat band.

where $d_j^\dagger$ (d_j) and $c_j^\dagger$ (c_j) are electron creation (annihilation) operators for the lattice and leads, respectively. $V_0/2$ is a hopping strength in the leads and g is a coupling strength between the cross-stitch lattice and leads.

$$E\phi_{-1} = -\frac{V_0}{2}\phi_{-2} - g a_1 \tag{S9}$$

$$E a_1 = H_0 a_1 + H_1 a_2 - g\phi_{-1} \tag{S10}$$

$$E a_j = H_0 a_j + H_1^\dagger a_{j-1} + H_1 a_{j+1} (2 \leq j \leq N-1) \tag{S11}$$

$$E a_N = H_0 a_N + H_1^\dagger a_{N-1} - g\phi_1 \tag{S12}$$

$$E\phi_1 = -\frac{V_0}{2}\phi_2 - g a_N, \tag{S13}$$

where

$$\phi_j = e^{iqj} + r_0 e^{-iqj} \quad (j < 0) \tag{S14}$$

$$= t_0 e^{iqj} \quad (j > 0). \tag{S15}$$

$|r_0|^2$ and $|t_0|^2$ are reflection and transmission probabilities, respectively, and $|r_0|^2 + |t_0|^2 = 1$ in Hermitian cases. Finally, we obtain the equations as follows

$$-\frac{V_0}{2} = \frac{V_0}{2} r_0 - g a_1 \tag{S16}$$

$$g e^{-iq} = -g e^{iq} r_0 + (H_0 - E) a_1 + H_1 a_2 \tag{S17}$$

$$0 = H_1^\dagger a_{j-1} + (H_0 - E) a_j + H_1 a_{j+1} \tag{S18}$$

$$0 = H_1^\dagger a_{N-1} + (H_0 - E) a_N - g e^{iq} t_0 \tag{S19}$$

$$0 = \frac{V_0}{2} t_0 - g a_N, \tag{S20}$$

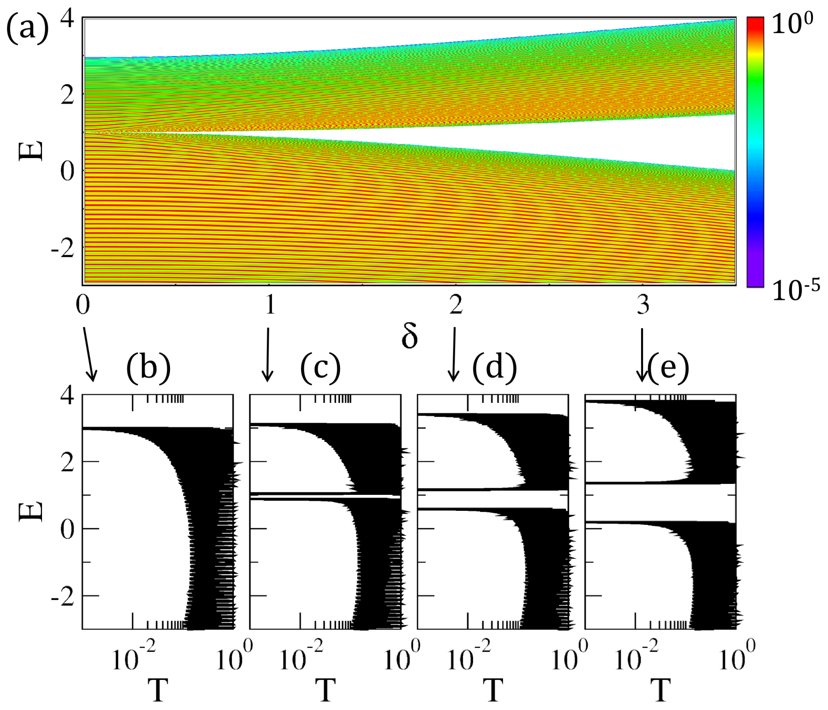

FIG. S2: (a) Transmission in (δ, E) space when $\gamma = 0$. Red curves represent high transmissions corresponding to the resonant modes in finite sized cross-stitch lattices. The transmission is smaller than 10^{-5} in the white region, which is a bandgap in transmission. The resonant states near the boundaries of the bands are not clear because of the high density of states. As δ increases, the sizes of the bands are mostly maintained, and the energy states related to the resonant modes do not disappear. (b-e) Transmissions when δ equals 0, 1, 2, and 3, respectively. As E approaches the boundaries of the bands, the spacings between transmission peaks decrease because of the high density of states.

where

$$e^{\pm iq} = -\frac{E}{V_0} \pm i\sqrt{1 - \left|\frac{E}{V_0}\right|^2}. \tag{S21}$$

Finally, we can obtain R and T from the following equation

$$\begin{pmatrix} -\frac{V_0}{2} \\ G_{2-} \\ 0 \\ \vdots \\ 0 \\ 0 \\ 0 \end{pmatrix} = \begin{pmatrix} \frac{V_0}{2} & G_1 & & & & & \\ G_{2+} & H_0 - EI & H_1 & & & & \\ & H_1^\dagger & H_0 - EI & H_1 & & & \\ & & \ddots & \ddots & \ddots & & \\ & & & H_1^\dagger & H_0 - EI & H_1 & \\ & & & & H_1^\dagger & H_0 - EI & G_{2+} \\ & & & & & G_1 & \frac{V_0}{2} \end{pmatrix} \begin{pmatrix} r_0 \\ a_1 \\ a_2 \\ \vdots \\ a_{N-1} \\ a_N \\ t_0 \end{pmatrix}, \tag{S22}$$

where

$$G_1 = -g \begin{pmatrix} 1 & 1 \end{pmatrix}, G_{2\pm} = \mp g e^{\pm iq} \begin{pmatrix} 1 \\ 1 \end{pmatrix}, \tag{S23}$$

and H_0 and H_1 are 2×2 matrices in the case of cross-stitch lattice with two leads coupled to both a- and b-sites of the end unit cells.

Figure S2 (a) shows the transmission in (δ, E) space when $\gamma = 0$. In the Hermitian case, resonant modes inside the bands do not disappear, so the energy bands almost maintain their widths and density of states, irrespective of perturbation strength. Figure S2 (b), (c), (d) and (e) are transmissions when δ equals 0, 1, 2, and 3, respectively.

III. EIGENENERGIES IN FINITE SIZED CROSS-STITCH LATTICES

We now obtain eigenenergies in finite sized cross-stitch lattices. From Eq. (S1) and Eq. (S2), H for the cross-stitch lattice with N unit cells is given by

$$H = \begin{pmatrix} \ddots & & & & \\ & H_0 & H_1 & & \\ & H_1^+ & H_0 & H_1 & \\ & & H_1^+ & H_0 & \\ & & & & \ddots \end{pmatrix}. \tag{S24}$$

Solving this $2N \times 2N$ matrix, we can obtain $2N$ eigenenergies. For instance, in the case of $\epsilon_a = \epsilon_b$, each band has N corresponding eigenenergies, i.e., N eigenenergies correspond to the flat band and N eigenenergies relate to the dispersion band.

IV. TRANSMISSION IN A NON-$\mathcal{PT}$-SYMMETRIC NON-HERMITIAN SYSTEM

Let us consider the transmission probability in a cross-stitch lattice with $\gamma = 1$ and different Γ. When $\Gamma = 0.1$, there are two peaks at $E_r \sim 0.54$ and $E_r \sim 1.47$ in transmission with real incident energy, which correspond to the transmission probability on the line of $E_i = 0.1$ when $\gamma = 1$ in Fig. S3 (b). When $\Gamma = 0.3$ and 0.5, there are also peaks in transmission with real incident energy, which correspond to the transmission probability on the lines of $E_i = 0.3$ and 0.5, respectively.

V. DETANGLING CROSS-STITCH LATTICES INTO FANO LATTICES

Following Ref. [S1], we can detangle $\mathcal{PT}$-symmetric cross-stitch lattices into Fano lattices. The amplitude equations for the Hamiltonian of the cross-stitch lattice (Eq. (S1)) are

$$Ea_n = \epsilon_n^a - da_{n+1} - da_{n-1} - db_{n+1} - db_{n-1} - tb_n, \tag{S25}$$
$$Eb_n = \epsilon_n^b - db_{n+1} - db_{n-1} - db_{n+1} - db_{n-1} - ta_n. \tag{S26}$$

If $\epsilon_a = \epsilon_b$, there is one flat and one dispersion band. From these equations, we obtain a 1D lattice with hopping strength $2d$, p_n, and side-coupled Fano states f_n,

$$Ep_n = (\epsilon_n^+ - t)p_n + \epsilon_n^{-1} f_n - 2d(p_{n+1} + p_{n-1}), \tag{S27}$$
$$Ef_n = (\epsilon_n^+ + t)f_n + \epsilon_n^{-1} p_n, \tag{S28}$$

where

$$p_n = \frac{1}{\sqrt{2}}(a_n + b_n), \qquad f_n = \frac{1}{\sqrt{2}}(a_n - b_n), \tag{S29}$$
$$\epsilon_n^+ = \frac{1}{2}(\epsilon_n^a + \epsilon_n^b), \qquad \epsilon_n^- = \frac{1}{2}(\epsilon_n^a - \epsilon_n^b). \tag{S30}$$

Figure S4 shows the Fano lattices detangled from a cross-stitch lattice. Horizontal couplings are $2d$ and vertical coupling ϵ_n^-. In the case of Hermitian perturbation, $\delta \neq 0$ and $\gamma = 0$, the real energy bands originate from the Hermitian (real value) coupling $\epsilon_n^- = \delta/2$ between the Fano states and 1D lattice. In the case of non-Hermitian perturbation, $\delta = 0$ and $\gamma \neq 0$, however, the complex energy bands originate from the non-Hermitian (imaginary value) coupling $\epsilon_n^- = i\gamma/2$ between the Fano states and 1D lattice. As a result, the difference between Hermitian and non-Hermitian perturbations depends on whether the couplings between Fano states and 1D lattice in the Fano lattices detangled from a cross-stitch lattice are real or imaginary.

[S1] S, Flach, D. Leykam, J. D. Bodyfelt, P. Matthies, A. S. Desyatnikov, Europhys. Lett. **105**, 30001 (2014).

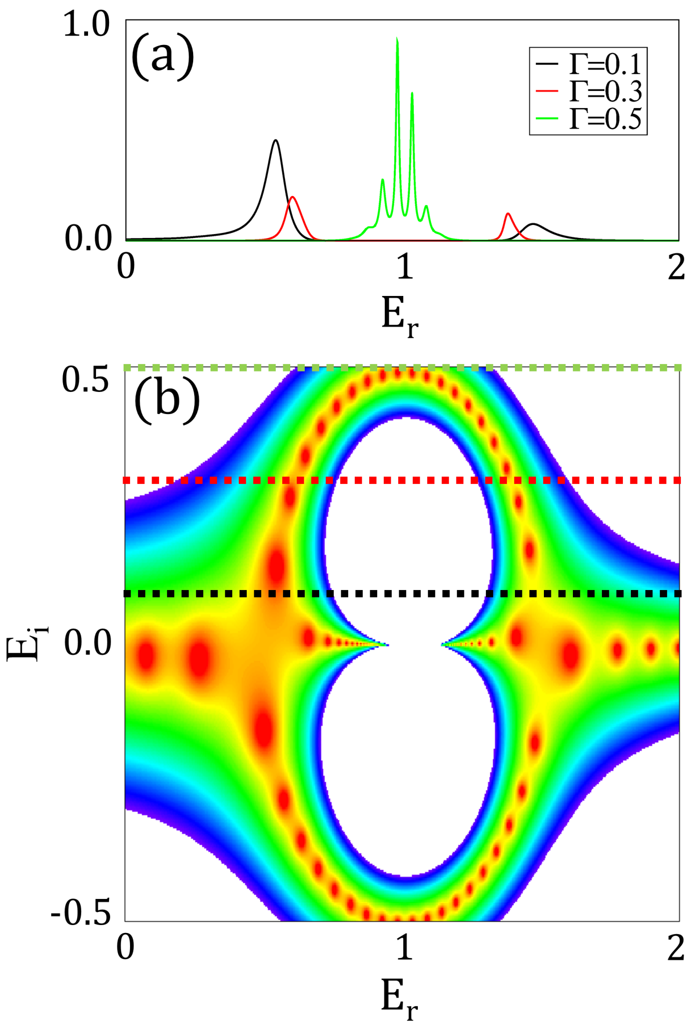

FIG. S3: (a) Transmission probabilities when $\Gamma = 0.1$ (black), 0.3 (red), and 0.5 (green). (b) Transmission probability on the complex incident energy plane when $\gamma = 1$. The red regions represent high transmission probability corresponding to the resonant states. The black, red, and green dotted lines represent $E_i = 0.1$, 0.3, and 0.5, respectively.

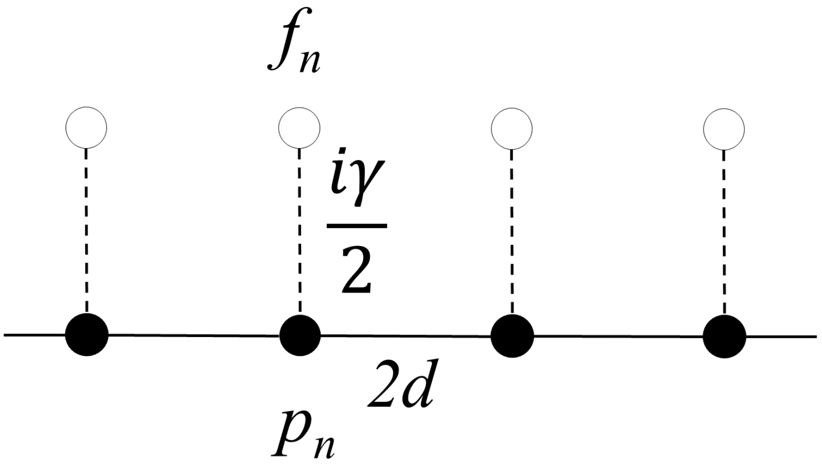

FIG. S4: Fano lattices detangled from a $\mathcal{PT}$-symmetric cross-stitch lattice.
